# Case report: lateral axillary-profunda femoris artery bypass for acute lower limb ischemia due to thrombosis after bilateral axillofemoral bypass

**DOI:** 10.1186/s13019-020-01232-w

**Published:** 2020-07-31

**Authors:** Kang She, Xiansheng Zhang, Jie Yin, Gong Cheng, Xiangrong Chen, Yufei Zhang

**Affiliations:** Vascular and Endovascular Surgery, Peking University First Hospital, 8 Xishuku Dajie, Xicheng District, Beijing, China

**Keywords:** Case report, Lateral axillary-profunda femoris artery bypass, Leriche syndrome, Complications, Acute lower limb ischemia

## Abstract

**Introduction:**

We treated a patient with late-stage unilateral bypass thrombosis after bilateral axillary-femoral bypass with lateral axillary-profunda femoris artery (LAx-PF) bypass.

**Case presentation:**

A 64-year-old male patient was admitted to our hospital for acute left lower limb ischemia. Six years ago, he underwent bilateral axillary-femoral bypass due to Leriche syndrome. On emergency admission, thrombosis of the left bypass vessel was identified. Blood flow could not be restored due to failure to restore patency of the proximal and distal anastomosis of the left bypass vessel during surgery. We performed LAx-PF bypass surgery to successfully rescue the limb, which was on the verge of necrosis.

**Conclusion:**

If thrombectomy cannot restore blood flow in the previous axillary-femoral bypass, LAx-PF bypass is an easy procedure to rescue the ischemic limb.

## Introduction

Axillofemoral bypass (axillary-femoral bypass) is usually used as one of the surgical methods for the treatment of Leriche syndrome and abdominal aortic aneurysms. It is an important extra-anatomical surgical approach and includes a variety of surgical procedures, such as axillary-unilateral femoral artery bypass, axillary-bilateral femoral bypass and bilateral axillary-femoral artery bypass [1]. Its patency rate is generally not superior to that of intra-anatomical abdominal aorto-bifemoral bypass, but it can play important roles in conditions such as a hostile abdomen and infectious abdominal aortic aneurysms [2–4]. Long-term complications after axillofemoral bypass include thrombosis, lower extremity arterial embolism and infection. Based on the urgency of symptoms and the cause and extent of thrombosis, limb ischemia caused by thrombosis after axillofemoral bypass can be treated by a variety of surgical methods, including thrombectomy of an artificial blood vessel, femoral artery patch angioplasty, and femoropopliteal bypass [1]. Lateral axillary-profunda femoris artery (LAx-PF) bypass, a non-routine bypass, has not been reported for the treatment of this type of complication.

## Case presentation

### Patient information

**Fig. 1:**
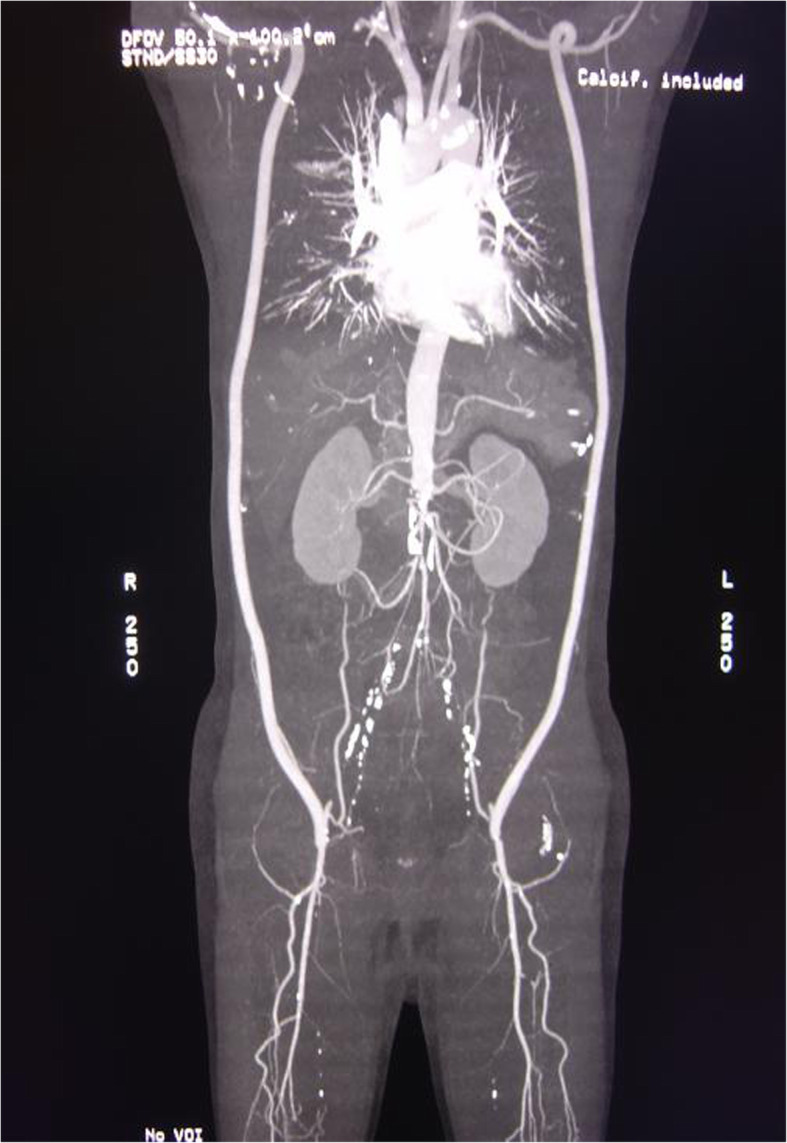
The patients previously underwent bilateral axillofemoral bypass due to Leriche syndrome. Postoperative CTA examination shows occlusion of the infrarenal abdominal aorta, bilateral internal iliac artery, external iliac artery, and bilateral superficial femoral artery. The deep femoral artery is well compensated, and the bilateral collateral circulation is unobstructed

A 64-year-old man was admitted to our hospital because of acute left lower limb ischemia. More than 6 years ago (December 2012), he was treated in our hospital for intermittent claudication, hip pain and impotence. Abdominal aorta angiography showed occlusion of the infrarenal abdominal aorta, bilateral common iliac artery, internal iliac arteries and bilateral superficial femoral arteries. He was diagnosed with Leriche syndrome type III [5]. The patient underwent bilateral axillofemoral bypass in December 2012. The bilateral axillary artery-artificial vessel anastomosis involved end-to-side anastomosis of an artificial vessel-second segment axillary artery, and the bilateral femoral artery anastomosis involved an end-to-side anastomosis of an artificial vessel to the femoral artery. His symptoms improved after surgery. He was able to walk more than 5 km and resumed sexual activity. Postoperative examination showed a pulse in the dorsalis pedis artery and posterior tibial artery of the bilateral lower limbs. Follow-up contrast-enhanced aortic computed tomography angiography (CTA) showed patency of bilateral axillary-femoral arterial bypass vessels (Fig. 1). The patient took warfarin orally after surgery. The international normalized ratio (INR) was adjusted to 1.5–2.5. He took hypolipidemic agents and vasodilator prostaglandins orally. Although his condition was controlled well by drug treatment, the patient did not quit smoking. In the most recent episode, the patient complained of numbness, coldness and intermittent claudication of the left lower limb after drinking for 4 days. The symptoms gradually worsened. The patient was admitted to the hospital in February 2019.

### Clinical findings

On admission, the patient’s vital signs were stable, and the blood pressure in both upper limbs was similar, 130/70 mmHg and 125/75 mmHg. The patient had no symptoms of ischemia in both upper limbs, and the pulses in both radial arteries were palpable. The skin color of the left lower limb was pale, and the skin temperature was significantly reduced, particularly in the lower leg. The muscle strength of the right lower limb was 3/5, and the pulses of the dorsalis pedis artery in the dorsal foot and posterior tibial arteries of the lower left limb were not palpable. The skin color, temperature and muscle strength of the right lower limb were normal, and the pulses of the dorsalis pedis artery and posterior tibial arteries of the right lower limb were palpable. The patient’s symptoms progressed rapidly after admission, including resting pain in the left lower extremity, and foot drop was present the day after admission. Routine preoperative examination showed a normal white blood cell count with an increased neutrophil percentage (86.4%). A biochemical panel showed that most parameters were within normal limits, except increased levels of glucose (9.87 mmol/L), creatine kinase (730 IU/L) and myoglobin (87.90 ng/ml). A coagulation test showed that when taking oral warfarin, the INR was 1.78, and the D-dimer level was increased (0.56 ng/L) (DDU). There were no significant electrocardiogram, echocardiogram, or chest X-ray abnormalities.

### Diagnostic assessment

Acute left lower limb ischemia can be easily diagnosed based on the patient’s 5P sign and laboratory examination. Additional diagnosis of Leriche syndrome type III, hypertension and diabetes was made based on previous medical history. Although acute ischemia of the left lower limb is likely to be caused by left bypass vascular thrombosis, the diagnosis of artificial vascular thrombosis is not based on objective examination. At the same time, considering the rapid progression of the patient’s symptoms and the presence of neuroischemia, we performed emergency arteriography to confirm further diagnosis and possible treatment.

**Fig. 2:**
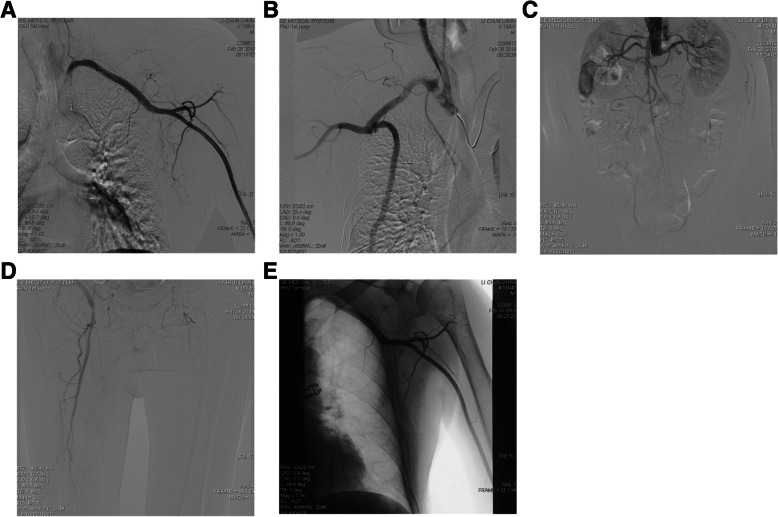
The patient’s preoperative angiography results: **a** The beginning segment of the left axillary-femoral bypass is occluded, and there is no blood flow in the artificial vessel. **b** No significant stenosis at the anastomosis of the right side axillofemoral bypass is evident. Smooth blood flow was noted through the artificial vessel. No stenosis is noted at the axillary artery anastomosis. **c** Occlusion of the abdominal aorta below the level of the renal artery, and occlusion of the common iliac artery, internal iliac artery and external iliac artery. **d** Occlusion of the superficial femoral artery in the bilateral lower limbs. The left common femoral artery anastomosis is not visible, and delayed contrast filling is noted in the deep femoral artery of the left lower extremity. There is no stenosis in the right lower limb femoral artery anastomosis, and the right deep femoral artery is unobstructed. **e** Attempts to use a guidewire catheter to access the left artificial vessel lumen were unsuccessful under angiography

The patient was in the supine position and under general anesthesia. First, we performed angiography of the bilateral branches of the aortic arch, abdominal aorta, and lower extremity arteries via the brachial artery of the left upper limb. The angiography showed no stenosis at the origin of the left subclavian artery, occlusion of the initial segment of the left axillofemoral bypass, and no blood flow in the artificial vessel (Fig. 2a). No stenosis was observed in the right brachiocephalic trunk and right subclavian artery. No significant stenosis was observed at the anastomosis of the axillofemoral bypass. Smooth blood flow was noted through the artificial blood vessel. No stenosis was observed at the axillary artery anastomosis (Fig. 2b). Occlusion of the abdominal aorta below the level of the renal artery and occlusion of the common iliac artery, internal iliac artery and external iliac artery were noted (Fig. 2c). Superficial femoral artery occlusion of both lower limbs was noted. The left common femoral artery anastomosis was not visible, and delayed contrast filling was noted in the deep femoral artery of the left lower extremity. There was no stenosis in the femoral artery anastomosis of the right lower limb, and the right deep femoral artery was unobstructed (Fig. 2d). We diagnosed the patient with thrombosis after left axillofemoral bypass.

### Therapeutic intervention

Initially, we tried to open the blocked graft with intracavitary intervention,but the attempts to use a guidewire catheter to access the left artificial vessel lumen was unsuccessful under angiography (Fig. 2e). A small incision was made in the left subclavian region to expose the artificial blood vessel in the path of the inflow vessel. A 5.5F double-lumen Forgarty catheter (Edward®) was used to perform thrombectomy at the proximal and distal ends. A purple-reddish thrombus (40 cm in length) was removed. However, repeated attempts failed to pass the thrombectomy catheter through the proximal artificial vessel-axillary artery anastomosis and the distal artificial vessel-femoral artery anastomosis. Because there was no blood flow in the inflow and outflow vessel of the original artificial blood vessel, the procedure was challenging.

**Fig. 3:**
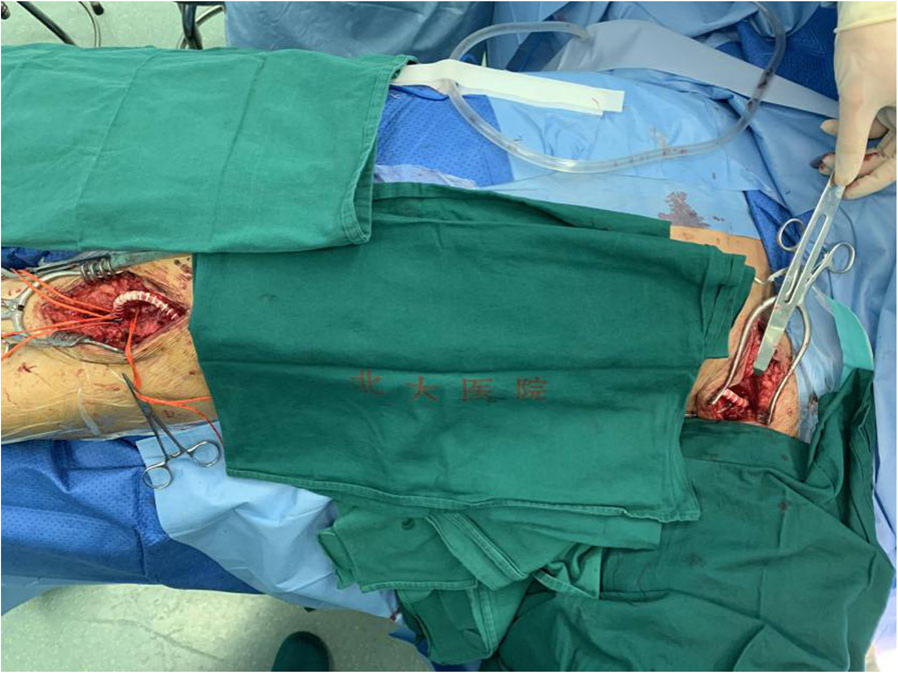
Left LAx-PF bypass

Fortunately, there was no obvious stenosis at the initial segment of the left subclavian artery in this patient, and intraoperative arterial blood pressure monitoring showed that the left upper limb arterial blood pressure was within normal limits. Therefore, we decided to use the third segment of the distal axillary artery as the inflow artery. The incision was extended to the axillary fossa to expose the left axillary artery. The axillary artery pulse was palpated very well. A longitudinal incision was made along the groin area of the left lower limb to fully expose the left lower limb artificial vessel-femoral artery anastomosis. The superficial femoral artery was occluded with a sclerotic plaque. We carefully exposed the left deep femoral artery and found no palpable sclerosis in the wall of the left femoral deep artery. We created a subcutaneous tunnel in the posterior axillary line in the thoracic and abdominal walls on the dorsal side of the original artificial blood vessel. The tunnel was turned at the anterior superior spine toward the anterior aspect of the thigh. After systemic heparinization, we clamped the left axillary artery, the left common femoral artery, the deep femoral artery and the superficial femoral artery. We introduced a 7 mm × 60 cm heparin-bonded vascular graft (GORE®PROPATEN®) into the subcutaneous tunnel and used CV-6 (GORE®) nonabsorbable sutures for end-to-side anastomoses between the artificial vessel and the axillary artery and the deep femoral artery (Fig. 3). After the clamp was removed, the deep femoral artery pulse at the distal end of the anastomosis was palpable. The incisions were closed in layers. The operative time was 3 h and 50 min, and the intraoperative blood loss was 80 ml.

After the surgery, the patient received anticoagulation, vasodilation and anti-infection treatment. The patient’s left lower extremity pain was significantly relieved. The left groin wound healed poorly after surgery due to lymph fluid leakage. However, there were no signs of infection such as fever and local swelling. The wound improved after periodical dressing changes.

### Follow-up and outcomes

**Fig. 4:**
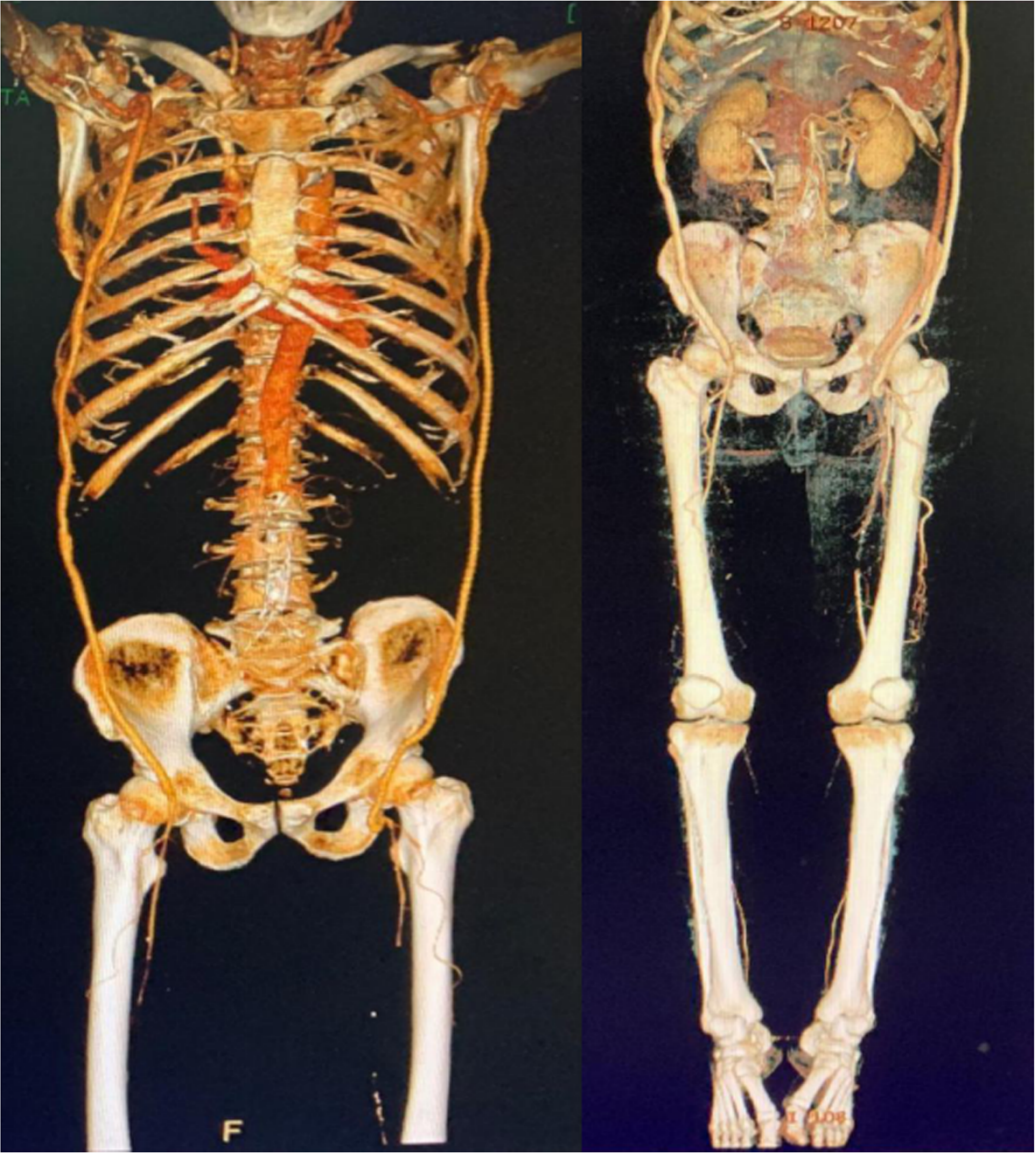
Postoperative CTA examination showing that the left LAx-PF bypass is unobstructed and that the previously occluded left axillary-femoral bypass vessel is visible. The right axillofemoral bypass is unobstructed

Postoperative follow-up enhanced CT showed that the left LAx-PF bypass was unobstructed, the original axillofemoral bypass had no blood flow, and the original right axillofemoral bypass was unobstructed (Fig. 4).

The patients continued to receive anticoagulation, vasodilation and lipid-lowering agents. During the one-year follow-up, no intermittent claudication and impotence were reported.

## Discussion and conclusions

Axillofemoral bypass is usually used as one of the surgical methods for the treatment of Leriche syndrome and abdominal aortic aneurysms. It is an important extra-anatomical surgical approach and includes a variety of surgical procedures such as axillary-unilateral femoral artery bypass, axillary-bilateral femoral bypass and bilateral axillary-femoral artery bypass [1]. Axillofemoral bypass was originally proposed by Blaisdell and Hall in 1963 [6]. If a patient has bilateral lesions, femorofemoral (femoral-femoral) bypass can be performed concurrently, that is, axillary-bifemoral artery bypass [7]. Its patency rate is generally not more ideal than that of intra-anatomical abdominal aorto-bifemoral bypass, but it can play important roles in conditions such as a hostile abdomen and infectious abdominal aortic aneurysms [2–4]. The patient underwent the prior procedure due to ischemic symptoms caused by Leriche syndrome. We discussed with the patient the benefits and risks of different surgical procedures including open surgery and interventional surgery. Because the patient’s abdominal aortic occlusion was at the level close to the renal artery, in order to avoid major trauma of open surgery, the high cost of interventional surgery and the risk of potential renal damage, we ultimately decided to perform bilateral axillofemoral bypass for surgical treatment. The procedure went smoothly. The patient recovered well after surgery with satisfactory patency of the bypass vessels.

The axillary artery is divided into the medial, middle and lateral segments by the pectoralis minor. In early studies, axillary-bifemoral artery bypass was created by using the proximal segment, that is, the axillary artery on the proximal edge of the pectoralis minor muscle, as the inflow vessel. In a subsequent study [1] and in our study, we used the middle segment, that is, the axillary artery behind the pectoralis minor muscle, as the inflow vessel. The advantages of using the middle segment of the axillary artery as the inflow vessel includes relatively easy exposure and a lower risk of brachial plexus injury. However, the use of the lateral axillary artery, that is, the axillary artery at the edge of the pectoralis major muscle and at the axillary fossa, as the inflow vessel for axillofemoral bypass has not been reported. In this case, we confirmed by angiography that there was no obvious stenosis from the left subclavicular artery orifices to the axillary artery, and the anastomosis between the original medial segment of axillary artery and the graft did not affect the diameter of the vessel. Therefore, it was reliable to use the lateral axillary artery as the inflow passage. In terms of the selection of the outflow vessel, the common femoral artery is always the best choice. Because an artificial blood vessel can play a similar role as a patch to widen the femoral artery, the blood flow in the bypass can directly supply the deep femoral artery and the superficial femoral artery simultaneously [1]. In cases where the superficial femoral artery is unobstructed or may be reestablished by endovasuclar treatment, the superficial femoral artery is still a better outflow vessel than the deep femoral artery. If the superficial femoral artery is occluded but the deep femoral artery is patent, lower extremity ischemia symptoms may be completely relieved. In this case, the patient had bilateral superficial femoral artery occlusion before the bilateral axillofemoral bypass was performed. However, the patient had no symptoms of lower limb ischemia and the pulses of the dorsalis pedis artery and posterior tibial artery of the bilateral lower limbs were palpable after surgery. This indicates that the blood flow of the deep formal artery is important and sufficient for lower extremity blood supply. Thus, during this procedure, we used the deep femoral artery as the outflow vessel without concerns. If the patient is considered to have insufficient blood flow when using the deep femoral artery as the outflow vessel, an additional femoral popliteal bypass or axillopopliteal bypass may be performed. Table 1 shows the indications and limitations of the lateral axillary-profunda femoris artery bypass.

**Table 1:** The indications, limitations and alternatives of the LAx-PF bypass

Indications			Limitations	Alternatives
Leriche syndrome;	**and**	Necessary to establish ananatomical bypass, but not suitable for the axillofemoral bypass or femorofemoral bypass;	The subclavian artery to the distal axillary artery must be free from definite stenosis or occlusion;	The more proximal inflow, such as the proximal segment axillary artery, the subclavian artery, or the aorta.
Abdominal aortic aneurysms;				
Abdominal aortic graft infection;				
		Axillofemoral bypass or femorofemoral bypass failure	The profunda artery is well compensated, as determined by symptoms, signs, or ABI;	Establish other outflow, such as reestablished superficial femoral artery, additional femoral popliteal bypass or axillopopliteal bypass.

Axillofemoral bypass can be performed under a variety of anesthesia, including general anesthesia, local anesthesia, regional anesthesia and even epidural anesthesia [8]. One of the reasons axillofemoral bypass was used to replace abdominal aorta-femoral artery bypass is that the risk of anesthesia is less in the former than that in the latter (open surgery). We chose general anesthesia for this procedure in this patient, and no obvious anesthesia complications and perioperative cardio-cerebral vascular accidents occurred. Based on the anesthesia experience from the arteriovenous fistula of the upper arm, LAx-PF bypass can be performed under local anesthesia to minimize the risk of general anesthesia for patients.

Complications after axillofemoral bypass include thrombosis, lower extremity arterial embolism and infection. Like other bypasses, the main complication of axillofemoral bypass is still postoperative thrombosis, which is often secondary to intimal hyperplasia of the distal anastomosis. For patients with bilateral lesions, such as Leriche syndrome, axillary-bilateral femoral bypass or axillofemoral bypass is an option. The patency rates of axillary-bifemoral artery bypass and axillary-unilateral femoral artery bypass are reported inconsistently. Early studies have shown that the patency after axillary-bifemoral artery bypass is higher, probably because the blood flow is bifurcated by two outflow vessels and reduces the impact on the distal artery of the anastomosis and thus reduces the degree of intimal hyperplasia [9]. Moreover, it is often secondary to intimal hyperplasia at the distal end of the anastomotic site [9]. Other causes of graft thrombosis may include anastomotic stenosis, inflow stenosis, and hypercoagulability. However, recent large-sample-sized studies have shown that the 1-year patency rates for axillary-bifemoral artery bypass and axillary-unilateral femoral artery bypass are similar [10]. Courbier et al. showed that the 5-year patency rate for axillary-bilateral formal artery bypass is between 30 and 85% [11]. A study by Blaisdell et al. showed that the 2-year patency rate for bilateral axillofemoral bypass was 75%, while that for unilateral axillofemoral bypass was 50% [12]. The long-term patency rates for bilateral axillofemoral bypass and axillary-bilateral femoral bypass remain unclear. Moreover, a study that included a small number of cases suggested that in some nonobstructive lesions, for instance, axillary-bilateral femoral bypass for the treatment of abdominal aortic aneurysms may not provide sufficient blood flow to the bilateral lower limbs, and thus bilateral axillofemoral bypass is needed [13]. In addition, there are different specific anastomosis methods for axillary-bifemoral artery bypass. Some surgeons anastomose the femoral-femoral bypass vessel to the autologous common femoral artery [6]. Others anastomose the femoral-femoral bypass vessel to the artificial vessel for axillofemoral bypass [14]. Obviously, if thrombus occurs in a patient undergoing axillary-bifemoral artery bypass, the thrombus may grow into the autologous and artificial blood vessels and result in simultaneous ischemia of the bilateral lower limbs [15]. If a patient undergoes bilateral axillary-femoral bypass, unilateral thrombosis will not affect the contralateral lower limb. Surgical treatment for acute ischemia of one limb is easier than that for bilateral limbs. Therefore, we use bilateral axillofemoral bypass instead of axillary-bifemoral artery bypass. This patient had a unilateral axillary-femoral artery bypass thrombosis, and angiography showed that the contralateral bypass was completely unobstructed with no significant anastomotic stenosis. Although we can solve left lower limb ischemia by performing right femoral-left femoral deep artery bypass, we still choose to use the left lateral axillary artery as the inflow vessel.

There are many surgical treatments for limb ischemia caused by delayed thrombosis after axillofemoral bypass. Different surgical methods can be chosen according to the symptoms, timing, location and scope of the thrombosis, including drug thrombolysis, thrombectomy of the artificial blood vessel or autologous vessel, patch angioplasty, distal bridging bypass and aortic-bifemoral bypass [1, 16–18]. Because most chronic thromboses are secondary to the narrowing of the outflow vessel anastomosis, maintaining patency of the outflow vessel is the focus for a successful procedure [1]. In this patient, the previous anastomosis was located at the femoral artery bifurcation, the original superficial femoral artery had been completely occluded for a long time, and the deep femoral artery was well compensated; therefore, we chose the deep femoral artery as the outflow vessel. However, we did not expect that the inflow vessel could not be used for thrombectomy to restore blood flow. One reason may be the severe stenosis of the inflow vessel due to anastomotic hyperplasia, and the other reason is that the anastomosis angle between the artificial vessel and axillary artery was closer to 90 degrees, which made the embolization catheter difficult to pass. Therefore, some researchers have proposed that the anastomotic angle between the axillary artery and the femoral artery should be more acute to thereby make the anastomosis relatively wider and to reduce the anastomosis stenosis and anastomotic tension caused by shoulder movement [1].

Due to advances in endovascular interventional surgical instruments and methods in the past 10 years, axillofemoral bypass as a surgical method to treat Leriche syndrome has gradually become useless. It is more often used as a backup surgery for infectious abdominal aortic aneurysms [19]. However, we still need to deal with the postoperative complications of this type of bypass surgery, such as thrombosis and infection. The LAx-PF bypass provides a simple and convenient surgical solution for the treatment of acute lower limb ischemia caused by axillary-femoral artery thrombosis.

We treated a patient with acute ischemia of the lower extremity due to thrombosis after bilateral axillofemoral bypass. Late-stage thrombosis after axillofemoral bypass can cause acute lower limb ischemia. If thrombectomy cannot restore blood flow in the previous bypass vessel, LAx-PF bypass is an easy procedure to rescue the ischemic limb.

## Data Availability

Not applicable.

## Abbreviations

LAx-PF: Lateral axillary-profunda femoris artery
CTA: Computed tomography angiography
ABI: Ankle brachial index
